# Socioeconomics and Major Disabilities: Characteristics of Working-Age Adults in Rwanda

**DOI:** 10.1371/journal.pone.0153741

**Published:** 2016-04-21

**Authors:** Joshua Kiregu, Nathalie K. Murindahabi, David Tumusiime, Dana R. Thomson, Bethany L. Hedt-Gauthier, Anita Ahayo

**Affiliations:** 1 University of Rwanda, College of Medicine and Health Sciences, Kigali, Rwanda; 2 Harvard Medical School, Department of Global Health and Social Medicine, Boston, MA, United States of America; 3 Rwanda Biomedical Centre, Injuries and Disabilities Unit, Kigali, Rwanda; University of Perugia, ITALY

## Abstract

**Background:**

Disability affects approximately 15% of the world’s population, and has adverse socio-economic effects, especially for the poor. In Rwanda, there are a number of government compensation programs that support the poor, but not specifically persons with disability (PWDs). This study investigates the relationship between poverty and government compensation on disability among working-age adults in Rwanda.

**Methods:**

This was a secondary analysis of 35,114 adults aged 16 to 65 interviewed in the 2010/2011 Rwanda Household Wealth and Living Conditions survey, a national cross-sectional two-stage cluster survey, stratified by district. This study estimated self-reported major disability, and used chi-square tests to estimate associations (p<0.1) with income, government compensation, occupation type, participation in public works programs, and household poverty status. Non-collinear economic variables were included in a multivariate logistic regression, along with socio-demographic confounders that modified the relationship between any economic predictor and the outcome by 10% or more. All analyses adjusted for sampling weights, stratification, and clustering of households.

**Results:**

Over 4% of working-age adults reported having a major disability and the most prevalent types of disability in order were physical, mental, and then sensory disability. In bivariate analysis, annual income, occupation type, and poverty status were associated with major disability (p<0.001 for all). Occupation type was dropped because it was collinear with income. Age, education, and urban/rural residence were confounders. In the multivariate analysis, adults in all income groups had about half the odds of disability compared to adults with no income (Rwf1-120,000 OR = 0.57; Rwf120,000–250,000 OR = 0.61; Rwf250,000–1,000,000 OR = 0.59; Rwf1,000,000+ OR = 0.66; p<0.05 for all), and non-poor adults had 0.77 the odds of disability compared to poor adults (p = 0.001).

**Conclusion:**

Given that personal income rather than government programming is associated with disability in Rwanda, we recommend deliberately targeted services to those with disability via cash transfers, placements in disability-appropriate employment, and micro-savings programs.

## Introduction

An estimated 1 billion individuals (15% of the population) worldwide have some form of physical, sensorial (vision and hearing), or mental-behavioral disability with a subset of 3% estimated to have severe disability [1]. Estimates of overall disability for ages 15–59 are higher in Africa (19%) than other low- and middle-income countries in Southeast Asia (16%), and the Americas (15%), though rates in Africa range from 13% in Ghana to 24% in South Africa [1]. Estimated severe disability does not vary much by region; Africa (3.1%), Southeast Asia (2.9%), and the Americas (2.6%) have similar rates [1]. The overall rate of disability in Rwanda as measured by the 2012 Census and the 2011 Integrated Household Living Conditions Survey (EICV3) is estimated to be around 5% [2], [3], which is substantially lower than neighboring countries, and severe disability is not well studied in this country.

Various diseases, injuries and disorders are known to cause disabilities [1], but the links are not always direct. Other contextual factors in the environment, for example high vehicle traffic, and individual factors such as working in dangerous jobs, can influence who becomes disabled [4]. Environmental and individual risks for disability are often not evenly distributed in the population, but rather clustered in poor communities and marginalized social groups [5]. In this way, disability has a bidirectional relationship with poverty where disability can cause poverty, and poverty can increase risk of disability [6]. Furthermore, with onset of disability, evidence suggests that employment and average income decrease [7]. In Rwanda, we expect to find severe disability among the most impoverished because they poor face more socio-economic and health risk factors for having disabilities, and because people with disabilities (PWDs) face more barriers than those without disability to exit poverty [8].

The Rwandan Government has an ambitious plan to achieve middle income status by the year 2020 through a variety of national programs targeting socioeconomic improvement. A flagship program of the Economic Development and Poverty Reduction Strategy (EDPRS 2) 2008–2012 is the Vision 2020 Umurenge Program (VUP) [9]. This integrated large-scale development program targets the poorest households with (i) direct unconditional cash transfers, (ii) paid public works jobs, and (iii) creating access to financial services for the poorest citizens. The direct cash support is provided to households with no adults able to participate in public works including the elderly, child-headed households, the chronically sick, lactating mothers and PWDs [10]. Select PWDs in Rwanda also have access to social protection programs such as the Rwanda Demobilization and Reintegration program that provides assistance to demobilized soldiers with disabilities, and the Genocide Survivors Support and Assistance Fund that provides cash, housing, healthcare, education and income generation support for genocide victims.

Social protection programs and cash income ensure that vulnerable groups living with disabilities are carried forward in the national journey out of poverty. However, it is unclear whether Rwandans with disabilities have access to, or are participating in, these activities. This paper analyzes self-reported severe disability in Rwanda’s adult population and investigates the relationship between disability and socioeconomic status. We specifically explore access to government compensation programs, and having cash income.

## Methods

### Study design and population

We had the option to use data from the 2012 Census or the 2010/2011 Integrated Household Living Conditions Survey (EICV3) for this analysis. The census asked broadly about activity limitations. We chose to use data from the EICV3 which asks “Do you suffer from a *major* disability which affects your life in general?” because this question captures the most severe cases of disability in Rwanda, and is therefore better suited to explore the relationship between severe disability with poverty and government compensation. Furthermore, the EICV3 included more economic variables to be tested as predictors.

The EICV3 is a national-representative cross-sectional household survey conducted every five years in Rwanda since 2000 to monitor poverty and living conditions. The participants are randomly selected using two-stage cluster sampling, stratified by district [11]. Enumeration areas from the 2002 Census were the primary sampling unit and households were the secondary sampling unit. The EICV3 survey collected data on 68,398 individuals residing in 14,308 households. This study was restricted to the 35,114 adults age 16 to 65 at the time of interview. Individuals under age 16 were excluded as Rwandan labour laws prohibits their engagement in economic activities, even as apprentices [12], and individuals above 65 years were excluded as that is the mandatory retirement age in Rwanda [13].

### Study setting

Rwanda’s physical landscape poses many barriers to those with physical and visual disabilities. Rwanda is a small landlocked country in central-east Africa with a geographical terrain dominated by mountains in the west and savannah in the east. Rwanda has a population of about 10.5 million inhabitants of whom 83.5% reside in rural areas [14]. Rwanda is also one of the most densely populated countries in Africa at 415 inhabitants per square kilometer; its capital city, Kigali, has 1,552 inhabitants per square kilometer [15]. While a national highway network is paved, as are a sizable number of roads in Kigali, most of the population resides by unpaved roads which become washed out during the two rainy seasons (September-November, February-April). Within Kigali, most main roads have elevated sidewalks, and the hills and open-air drainage systems that intersect sidewalks create barriers for pedestrians with physical or visual disabilities.

Rwanda’s 1994 genocide brought about the brutal murder of up to 1 million people [16], [17], and left scores disfigured and psychologically scarred [18], [19]. Unpublished results from EICV3 show that of people with major disability, 5.6% self-report disability due to the Genocide. The annual Genocide commemoration coincides with special mental health services at events and advocacy campaigns to utilize existing psychosocial services at health facilities. Despite these efforts, Rwanda faces many of the same barriers in mental health as other low-resource countries including lack of internationally-recognized mental health indicators in the Millennium Development Goals (MDGs) and other international metrics, insufficient funding, and understaffing of mental health professionals [20].

### Data collection and analysis

The primary outcome is presence of any major disability “….which affects your life in general.” The EICV3 also collected data on the “main disability suffered” categorized by the data collector as visual disability, deaf and/or mute, disability in the arms, disability in the legs, mental disability, trauma, very old, and others.

Five indicators of socioeconomic wellbeing were assessed. Income and government compensation were each measured in Rwandan Francs received per year and categorized into five income categories. Occupation was grouped into four categories: professionals and senior managers, skilled services (which included office clerks, commercial and sales, skilled service sector), agricultural and fishery workers, and semi-skilled operators and unskilled labourers. Respondents’ participation in VUP in the last year were dichotomized. Poverty status was categorized by the EICV3 into non-poor, poor, and extremely poor, where poor are people who live under the national poverty line (RwF 118,000/US$ 205 per year in 2010 terms) and extremely poor are people who live on less than RwF 83,000/US$145 per year [21].

We tested bivariate associations between major disability and demographic and socio-economic characteristics using chi-square tests. Only those variables that were associated with major disability at p<0.1 were advanced for multivariable regression modelling. In the multivariate model, we adjusted for any socio-demographic confounders that changed the co-efficient measuring the relationship between disability and socioeconomic by 10% or more. We considered those economic variables associated at α<0.05 to be statistically significant, and we report odds ratios, p-values, and 95% confidence intervals. All analyses were performed in Stata version 13, and adjusted for sampling weights, stratification, and clustering of households.

### Ethics statement

This study is a secondary analysis of the EICV3 dataset which we acquired by requesting online access via the National Institute of Statistics in Rwanda (NISR). In this survey, informed verbal consent was obtained from every participant after reading a consent statement and before the start of the interview. Verbal consent was obtained rather than written consent to provide respondents an opportunity to ask questions, because many respondents were not literate, and because all data are collected via verbal interview. Consent was recorded by the interviewer. The EICV3, including this consent procedure, received ethical approval from the National Institute of Statistics, and ethical review for this analysis was provided by the University of Rwanda-College of Medicine and Health Sciences Internal Review Board. The dataset is de-identified, and we made every effort to ensure our analysis did not create the possibility of identifying persons surveyed.

## Results

Of the 35,114 individuals age 16 to 65 years in the EICV3, 1,513 (4.3%) self-reported major disability. Of the population with self-reported major disability, physical disability was most common (53.2%), followed by mental disability (28.8%) and sensory disability (18.0%) (Table 1). Descriptive analysis showed that major disability was twice as common in rural areas (4.4%) than in urban areas (2.9%), with the highest rates in Southern (5.3%) and Western (4.7%) provinces (Table 1). Also, major disability was less common among people with secondary (1.8%) and bachelors or higher (0.1%) education, compared to those with no formal education (7.7%) (Table 1). In bivariate analysis of economic factors and major disability, annual income, occupation type, and poverty status were associated with self-reported major disability (p<0.001 for all) (Table 2). Government compensation and having VUP were not independently associated with major disability.

**Table 1:** Socio-Demographic Characteristics of Working-age Adults with Self-Reported Disabilities in Rwanda.

				Disability Categories					
	Total (N)	With Disabilities (n)	%	Physical (n)	%	Sensory (n)	%	Mental (n)	%
**Province**									
Kigali City	3,890	84	(2.2)	57	(1.6)	8	(0.2)	19	(0.4)
Southern	9,085	507	(5.5)	223	(2.4)	106	(1.2)	178	(1.9)
Western	8,339	389	(4.8)	246	(3.0)	55	(0.7)	88	(1.1)
Northern	5,746	228	(3.9)	123	(2.1)	59	(1.1)	46	(0.7)
Eastern	8,054	305	(3.7)	160	(2.1)	43	(0.5)	102	(1.1)
**Urban/Rural**									
Urban	5,982	175	(2.9)	91	(1.6)	23	(0.4)	61	(0.9)
Rural	29,132	1,338	(4.5)	718	(2.4)	248	(0.9)	372	(1.2)
**Sex**									
Female	16,223	748	(4.0)	419	(2.0)	136	(0.8)	193	(1.2)
Male	18,891	765	(4.4)	390	(2.5)	135	(0.8)	240	(1.2)
**Age in Years**									
16–25	13,683	373	(2.7)	155	(1.2)	83	(0.6)	135	(0.9)
26–35	9,171	306	(3.1)	153	(1.6)	61	(0.6)	92	(0.9)
36–45	5,545	263	(4.6)	146	(2.7)	32	(0.5)	85	(1.4)
46–55	4,294	312	(7.4)	188	(4.4)	52	(1.3)	72	(1.7)
56–65	2,421	259	(10.5)	167	(6.7)	43	(2.0)	49	(1.9)
**Education**									
No formal education	5,798	444	(7.6)	214	(3.7)	89	(1.6)	141	(2.3)
Less than primary	15,809	701	(4.4)	371	(2.3)	124	(0.8)	206	(1.2)
Primary	10,320	318	(3.0)	190	(1.8)	49	(0.4)	79	(0.7)
Secondary	2,557	43	(1.8)	28	(1.3)	8	(0.3)	7	(0.2)
Bachelor’s or higher	573	7	(1.0)	6	(0.9)	1	(0.1)	0	(0.0)
Missing	57	0	(0.0)	0	(0.0)	0	(0.0)	0	(0.0)
**Total**	**35,114**	**1,513**	**(4.3)**	**809**	**(2.3)**	**271**	**(0.8)**	**433**	**(1.1)**

**Table 2:** Bivariate Associations Between Socio-Economic Factors and Major Disability in Rwanda, 2011.

	Total (N)	Major Disability (n)	(%)	95% C.I.	p-value
Economic Factors					
**Annual income in categories (RWF/Year)**					**<0.001**
No job / In-kind compensation	17,993	937	5.1	[4.8,5.4]	
1–120,000	2,398	73	3.0	[2.4,3.8]	
120,001–250,000	6,559	268	4.1	[3.5,4.8]	
250,001–1,000,000	6,676	201	3.1	[2.6,3.6]	
1,000,000 +	1,488	34	2.2	[1.5,3.0]	
**Total**	**35,114**	**1,513**			
**Govt. compensation in categories (RWF/Year)**					**0.116**
No compensation	2,710	114	4.4	[3.6,5.5]	
1–12,000	19,044	798	4.1	[3.8,4.5]	
12,001–24,000	2,657	135	5.3	[4.4,6.4]	
24,001–48,000	2,194	110	4.7	[3.9,5.8]	
48,000 +	8,509	356	4.1	[3.6,4.6]	
**Total**	**35,114**	**1,513**			
**Occupation type** *					**<0.001**
Professional & senior officers	1,083	20	1.6	[1.0,2.6]	
Commercial, skilled service & clerks	7,607	211	2.6	[2.3,3.0]	
Agricultural & fishery workers	19,744	909	4.7	[4.3,5.1]	
Semi-skilled operators & unskilled laborers	1,086	33	2.9	[2.0,4.1]	
No occupation: Sickness, Old age, Disability	311	232	70.3	[64.7,75.3]	
No occupation: Student, retired, Other	4,852	125	2.3	[1.8,2.9]	
**Total**	**34,683**	**1,507**			
Participated in VUP public works program last 12 months *					**0.369**
Yes	574	27	4.8	[3.4,6.8]	
No	8,440	389	4.5	[4.0,5.0]	
VUP does not exist here	26,088	1,096	4.2	[3.9,4.5]	
**Total**	**35,102**	**1,512**			
**Poverty status**					**<0.001**
Extremely poor	7,225	398	5.4	[4.8,6.0]	
Poor	6,841	331	4.8	[4.2,5.4]	
Non-poor	21,048	784	3.7	[3.4,4.0]	
**Total**	**35,114**	**1,536**			

* Missing 431 responses to “Occupation type” and 12 responses to “Participated in VUP public works program last 12 months”

In the multivariable model, having any amount of annual income was associated with lower disability (Table 3). Disability was lower among those who earn RwF 1–120,000 (US$0.01–209) (OR = 0.57, p<0.001), RwF 120,000–250,000 (US$209–435) (OR = 0.61, p<0.001), RwF 250,000–1,000,000 (US$435–1,740) (OR = 0.59, p<0.001), and RwF 1,000,000+ (US$1,741+) (OR = 0.66, p = 0.028) compared to those with no income. Also, the non-poor had 0.77 the odds of self-reported major disability compared to the poor (p = 0.001). Government compensation and participating in VUP activities remained non-significant in the multivariate model.

**Table 3:** Multivariate Logistic Regression Model With Odds Ratio, P-Value and Confidence Interval of People with Self Reported Major Disability*.

	OR	p-value	95% C.I.
**Annual Income**			
No job / In-kind compensation	1.00		
1–120,000	0.57	<0.001	[0.44,0.73]
120,001–250,000	0.61	<0.001	[0.50,0.73]
250,001–1,000,000	0.59	<0.001	[0.49,0.70]
1,000,000 +	0.66	0.028	[0.45,0.96]
**Government compensation**			
No compensation	1.00		
1–12,000 RwF	0.86	0.203	[0.68,1.09]
12,001–24,000 RwF	1.12	0.457	[0.83,1.51]
24,001–48,000 RwF	1.03	0.870	[0.75,1.40]
48,000 + RwF	1.00	0.998	[0.77,1.29]
**VUP**			
Yes	1.00		
No	1.11	0.619	[0.74,1.65]
VUP does not exist here	0.95	0.815	[0.64,1.41]
**Poverty status**			
Extremely poor	1.00		
Poor	0.88	0.194	[0.73,1.07]
Non-poor	0.77	0.001	[0.66,0.90]

*Adjusting for: education, age and urban-rural residence

## Discussion

This study found a 4.2% prevalence of self-reported major disability among working-age adults in Rwanda. This is consistent with data collected in Zambia and South Africa using different but comparable measures. In those countries, approximately 2% of the population had complete inability in one or more disability domains, and between 6% and 8% of the population had a lot of difficulty or complete inability in one or more disability domains [1], [22], [23].

Our finding that Southern and Western provinces had higher rates of disability than other provinces was also found in a less methodologically rigorous 2011 disability assessment in Rwanda [23]. The authors of that report suggest this could be due to the provinces hosting centers and schools for disabled persons which may attract people with disabilities and their families. This geographic disparity, along with the substantial difference in major disability between Rwanda and neighboring countries, warrants further investigation. Furthermore, our results suggest an association between disability and income, but not necessarily between disability and social protection programs, which we discuss next.

### Income & Poverty

In this study, having any income was associated with lower disability rates. The 2011 poverty line in Rwanda was quite low (118,000 RwF/US$ 205 per annum), reflecting the fact that 85% of Rwandans are subsistence farmers [24]. Onset of disability significantly limits income due to job loss and inability to get employment [25], and the more adverse the disability, the greater the effect [26]. Not only does exclusion of people with disabilities from the workforce limit household income, it can result in dependence on others, lower quality of life and burden other household members. A 2005 study in Rwanda found that people with lower limb amputations had great difficulty getting exercise and had high rates of alcohol and drug abuse [27]. In 9 out of 15 developing countries, employment rates are lower for people with disabilities, though there is great variation in the gap, ranging from 4% in Malawi, to 40% in Peru. Low income countries have lesser variability in disability gap as compared to middle income countries, suggesting that people living with disabilities could face more barriers as countries develop [28].

We looked at the relationship between disability and multiple levels of income. We did not find any indication that disability decreases as income rises. The lack of difference of disability across income groups may reflect limited differences in life-style and life experience across income levels, as the lowest income group (1–120,000 RwF) earns the equivalent of up to US$17 per month and the highest income group (1,000,000+ RwF) earns up to US$145 or more per month, which are both very low income. This is consistent with our other finding that any income, and therefore any participation in the workforce, is associated with lower disability. Other studies show that employment for people with disabilities significantly increases household income as compared to employment for people without disabilities [29], and therefore finding any employment opportunity for people with disabilities could heed positive impacts for the household.

### Government Compensation

Our results show no association between receiving government compensation and prevalence of disability. This may be because people with disability are not the only target of the VUP program [9]. Cash transfers to people who cannot work is just one aspect of the program; a major portion of people receiving VUP support are paid to perform labor intensive public works jobs. The EICV3 did not specify which of the three types of VUP support the respondent received (cash transfer, public works, and access to credit), therefore we were not able to model cash transfer alone.

In other countries, government and non-governmental organizations provide training and/or grants to people with disabilities [30], [31], which encourage participation in the labor force and socio-economic independence [32]. However, these kinds of grants usually do not protect people with disability against major shocks like a medical event or job loss [33]. Supplemental micro-saving programs can help to buffer this situation allowing people with disability to exit poverty [34]. Rwanda should similarly consider extending programs such as unconditional cash transfers targeting PWDs, as well as setting up special funds to support them to start businesses.

### Limitations

This study had several limitations. First, the measure of disability used in EICV3, self-reported “major disability”, is a less reliable measure of disability and results in lower estimates as compared to asking about broader functional limitations [35]. This is because the word “disability” is often understood to mean a permanent and unchangeable condition, often physical, where one can do almost nothing [36]. The 2001 WHO ICF framework [37] recommends posing questions about functional limitations rather than asking directly about disability because disability is contextually dependent; a person with relatively minor physical impairments might face major limitations without community infrastructure such as sidewalks and financial or medical resources like crutches. Recent conflict or other wide-spread adversity may further influence perceptions of disability across a community or nation.

Several other sources of self-reported overall disability are lower in Rwanda [2], [23] than in similar countries [7], [38], [39]. Although they come from different data sources with different questions, our estimate of “major disability” (4.2%) was close to the Rwanda 2012 Census estimate of 5% overall disability gathered with the broad activity limitation question: “do you have difficulty with seeing, hearing, speaking, walking or climbing, learning or concentrating or any other disability?”[3]. We offer several hypotheses about why overall self-reported disability is low in Rwanda, and why major disability accounts for such a large portion of overall disability.

One possible explanation is that Rwandans experience a strong individual-level sense of resilience to adversity that is reinforced by a strong national narrative of self-determination and transformation following the genocide [40]. Another explanation is that Rwandans enjoy stronger social support networks or more enabling physical environments than other nations leading to a higher level of functioning [41], though we believe this is unlikely as we are not able to find evidence of stronger social support networks [42] or better infrastructure [43] in Rwanda. Given the potential difference in how disability is perceived across nations, we recommend that future EICV surveys switch from direct questions about disability to the more technical and expensive ICF framework of functional limitations since these measures are more robust. Although we present hypotheses for unexpected difference in reported disability, the reasons are unclear and warrant further study.

A second limitation is that due to the cross-sectional nature of this study, we are not able to draw causal conclusions. We set up this analysis such that poverty “predicted” disability, though disability could have also caused poverty. Whether disability causes poverty, or poverty causes disability, our study shows a strong link between these phenomena, and highlights the need for targeted economic programs for the people with disability.

## Conclusions

Our results suggest that receipt of any income is important for PWDs, therefore we recommend that Rwanda’s government and governments in other similar settings deliberately target PWD with cash transfer programs and disability-appropriate employment. Supplemental micro-saving programs for this group should also be considered by governments and the private sector to help these households protect against shocks that perpetuate the cycle of poverty.
